# The mediating effect of depression on the association of hearing loss with all-cause and cardiovascular mortality: a prospective cohort study

**DOI:** 10.1186/s12889-025-25489-9

**Published:** 2025-11-25

**Authors:** Shaoyan Zhang, Haohong Lai, Chunling Liu

**Affiliations:** 1 Department of Otorhinolaryngology Head and Neck Surgery, the People’s Hospital of Baoan Shenzhen, 118 Longjinger Road, Shenzhen, Guangdong 518101 China; 2 Department of Otolaryngology, Sun Yat-sen Memorial Hospital, Sun Yat-sen University, 107th Yanjiangxi Road, Guangzhou, Guangdong 510120 China

**Keywords:** Depression, Hearing loss, NHANES, Mortality, Mediation analysis

## Abstract

**Background:**

Depression has been hypothesized to act as a mediator linking hearing loss to mortality; however, existing evidence remains limited and inconsistent. We aimed to investigate the potential mediating role of depression on the association of hearing loss with all-cause and cardiovascular mortality using a large, nationally representative prospective cohort of the U.S. population.

**Methods:**

This cohort study included 9,827 adults from the 2005–2018 National Health and Nutrition Examination Survey (NHANES). Multivariable Cox proportional hazards models were applied to examine the relationships of hearing loss and depression with all-cause and cardiovascular mortality. Multivariable linear regression was conducted to assess the link between hearing loss and depression. Mediation analysis was conducted to explore the mediating role of depression in the link between hearing loss and mortality outcomes.

**Results:**

After adjusting for confounders, both hearing loss and depression showed positive associations with all-cause and cardiovascular mortality, as well as with each other. Mediation analyses revealed that depression mediated the associations between speech-frequency, low-frequency, and high-frequency hearing loss and all-cause mortality by 10.28%, 10.15%, and 9.27%, respectively. For cardiovascular mortality, depression accounted for 11.40%, 11.25%, and 11.67% of the associations, respectively. Significant and stable mediation effects were also observed across various hearing frequency ranges (0.5–8 kHz).

**Conclusion:**

These findings underscore the substantial impact of depression on all-cause and cardiovascular mortality risks in individuals with hearing loss. Integrating routine depression screening and targeted mental health interventions into hearing loss management strategies is crucial to mitigating mortality outcomes in this population.

**Supplementary Information:**

The online version contains supplementary material available at 10.1186/s12889-025-25489-9.

## Background

Hearing loss is a common audiological condition impacting approximately 1.5 billion individuals globally, with projections estimating an increase to 2.5 billion by 2050 [1]. As the global population ages, hearing loss is now the third major contributor to years lived with disability, representing a pressing public health issue [2, 3]. Beyond its impact on auditory function, hearing loss significantly diminishes quality of life and poses considerable risks to mental health [4, 5]. Extensive research has demonstrated a strong link between hearing impairment and elevated rates of depression, underscoring the psychosocial burden associated with hearing loss [6–8]. Beyond its psychological consequences, hearing loss has also been linked to elevated all-cause and cardiovascular mortality, further stressing its public health importance [9–12]. While the underlying mechanisms connecting hearing loss to mortality remain unclear, depression has been postulated as a potential mediating factor [13]. Significantly, the connection between depression and increased mortality, both all-cause and cardiovascular, is well-documented [14–16]. However, the intricate interplay between hearing loss, depression, and mortality remains insufficiently explored, warranting further investigation.

Despite the recognition of these associations, limited research has assessed the mediating role of depression in the link between hearing loss and mortality. Appollonio et al. initially suggested that psychosocial factors, including mood and social relationships, mediate the connection between self-reported hearing loss and mortality [13]. However, their subsequent study concluded that the effect of sensory aid usage on mortality is primarily mediated through overall physical health and social relationships, rather than mood status [17]. Karpa et al. found that walking disability and cognitive impairment served as primary mediators between hearing loss and mortality, yet depression was not specifically assessed for its mediating role [18]. Genther et al. showed that the link between hearing impairment and mortality was marginally attenuated after adjusting for gait speed and cognition but remained unchanged after adjusting for depression, suggesting an uncertain role of depression as a mediator [19]. Xu and Francis found that psychological distress mediated the linkage of self-reported hearing impairment to cardiovascular disease but did not establish a direct link to cardiovascular mortality [20]. Miyawaki et al. conducted a mediation analysis and observed that depression partially contributed to the linkage of self-reported hearing loss to all-cause mortality, though the effect did not reach statistical significance [21]. These inconsistent findings, coupled with variations in study populations and lack of pure tone audiometry, highlight the need for more rigorous research to elucidate the role of depression in the link between hearing loss and mortality.

Given these gaps in the literature, it is crucial to clarify the mediating role of depression on the association of hearing loss with all-cause and cardiovascular mortality. To address this, we performed a large-scale prospective cohort study utilizing data from the National Health and Nutrition Examination Survey (NHANES) spanning 2005 to 2018. Furthermore, our research provides a more comprehensive examination of the mediating effect of depression across multiple hearing loss types, including speech-, low-, and high-frequency hearing loss, as well as hearing loss evaluated at seven frequencies (0.5, 1, 2, 3, 4, 6, and 8 kHz). By offering a more nuanced understanding of these interrelationships, our study aims to elucidate the mediating role of depression in the link between hearing loss and mortality, thereby providing empirical evidence for future research on hearing loss, mental health, and mortality.

## Methods

### Study population

Utilizing a rigorous four-stage stratified sampling design, NHANES is a nationwide program that collects nationally representative health and nutrition data from the civilian, non-institutionalized U.S. population [22]. NHANES gathers extensive information through structured interviews, physical examinations, and laboratory testing. All protocols were approved by the National Center for Health Statistics (NCHS) Ethics Review Board, and written informed consent was obtained from all participants. Detailed descriptions of the study design, methodology, and data collection procedures are available on the NHANES official website.

**Fig. 1:**
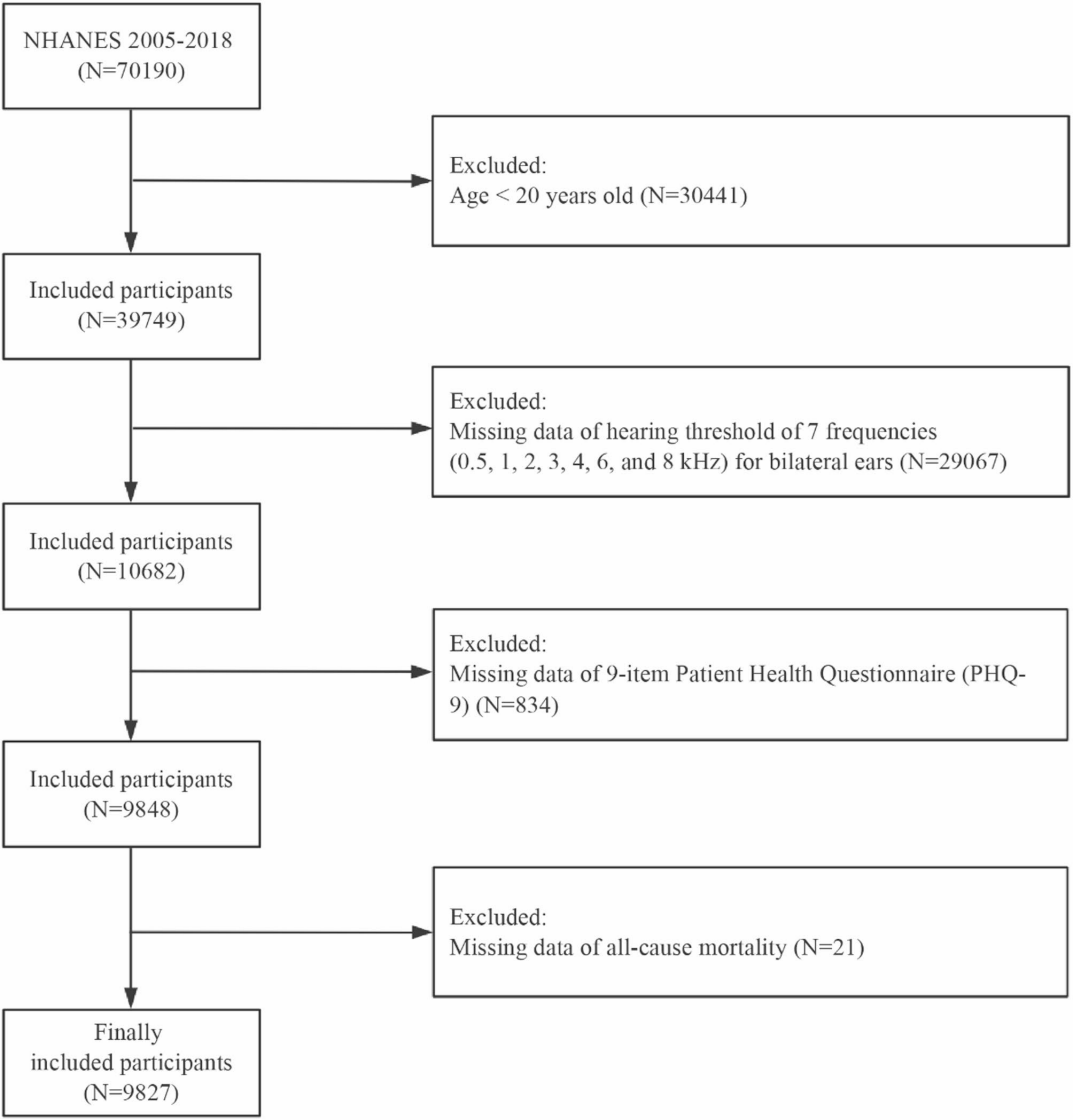
Flow diagram of the selection process for eligible participants. Abbreviations: NHANES, National Health and Nutrition Examination Survey

This prospective cohort study utilized data from the 2005–2018 NHANES. An initial sample of 70,190 participants was identified. Given that previous research has primarily focused on adults, and considering the greater prevalence and clinical relevance of both hearing loss and depression in this population, we restricted our analysis to adults (aged ≥ 20 years) to ensure comparability, enhance interpretability, and maintain sufficient statistical power, as mortality—particularly cardiovascular mortality—is rare among younger individuals. The following exclusion criteria were applied sequentially: (1) participants under 20 years old (*N* = 30,441); (2) participants with incomplete hearing threshold data for both ears (*N* = 29,067); (3) participants without 9-item Patient Health Questionnaire (PHQ-9) data (*N* = 834); and (4) participants lacking all-cause mortality data (*N* = 21). After exclusions, 9,827 eligible participants remained in the final analytical cohort, among whom 1,144 died from all causes during follow-up. Figure 1 presents a detailed flowchart of the participant selection process.

### Hearing assessment

Hearing assessments were conducted using pure-tone air conduction audiometry, administered by trained examiners in a sound-isolated room within the mobile examination center. Bilateral hearing thresholds were measured at seven frequencies (0.5, 1, 2, 3, 4, 6, and 8 kHz) using calibrated audiometers equipped with standard insert earphones and headphones. The intensity range of testing spanned from − 10 to 120 decibels (dB). For measurement reliability, hearing thresholds at 1 kHz were tested twice in both ears, and results were considered valid only if the difference between the two measurements did not exceed 10 dB. Detailed audiometric procedures are documented in the NHANES Audiometry Procedures Manual. For analysis, the speech-frequency pure-tone average (SF-PTA) was calculated by averaging hearing thresholds at 0.5, 1, 2, and 4 kHz. Similarly, the low-frequency pure-tone average (LF-PTA) was defined by averaging hearing thresholds at 0.5, 1, and 2 kHz, while the high-frequency pure-tone average (HF-PTA) was defined using thresholds at 3, 4, and 6 kHz. For each participant, the better-hearing ear, determined by the lower PTA, was selected as the hearing index for subsequent analyses. Notably, hearing was not consistently assessed during all NHANES waves for all participants. In our study, we included only adult participants (aged ≥ 20 years) with complete bilateral hearing threshold data. Specifically, we selected individuals who underwent pure-tone audiometry in the following NHANES cycles: 2005–2006 (aged ≥ 70 years), 2009–2010 (≥ 70 years), 2011–2012 (20–69 years), 2015–2016 (20–69 years), and 2017–2018 (≥ 70 years). Accordingly, our final analytical sample and the corresponding application of survey weights were restricted to adults aged ≥ 20 years from these specific cycles.

### Measurement of depressive symptoms

Depressive symptoms were evaluated using the PHQ-9, a well-validated self-reported screening questionnaire designed to assess the severity of depressive symptoms over the preceding two weeks [23, 24]. The PHQ-9 was administered via face-to-face, computer-assisted personal interviews to ensure standardized data collection. The questionnaire assesses nine core symptoms of depression, including depressed mood, changes in appetite, anhedonia, sleep disturbances, low self-esteem or feelings of worthlessness, concentration difficulties, fatigue, psychomotor agitation or retardation, and suicidal ideation [23]. Each question is rated on a scale of 0 to 3, producing a total score of 0 to 27, where higher values signify greater depressive symptom severity.

### Outcomes and follow-up

All-cause and cardiovascular mortality served as the primary endpoints. Mortality information was sourced from the 2019 Public-Use Linked Mortality Files provided by the NCHS (https://www.cdc.gov/nchs/data-linkage/mortality-public.htm). Follow-up started from each participant’s NHANES interview date and continued until December 31, 2019, or death, whichever came first. Follow-up time was defined as the total number of person-months contributed by each participant. The classification of death causes followed the International Classification of Diseases, 10th Revision (ICD-10). Cardiovascular mortality included deaths resulting from cerebrovascular or heart diseases, while all-cause mortality covered deaths from any cause.

### Covariates

The analysis adjusted for several potential confounding variables, including age, gender, race, body mass index (BMI), smoking and drinking status, as well as histories of hypertension, diabetes, and cardiovascular disease (CVD). Covariates were selected based on previous literature and their potential associations with hearing loss, depression, and mortality. All covariates were measured at the time of hearing assessment. Age was treated as a continuous variable in years. Sex was categorized as male or female. Race was grouped as Non-Hispanic White, Non-Hispanic Black, Mexican American, Other Hispanic, or Other (including multiracial). BMI was calculated as weight in kilograms divided by height in meters squared and analyzed as a continuous variable. Smoking status was categorized as non-smokers, former smokers, or current smokers according to their answers to two questions: “Have you smoked at least 100 cigarettes in your entire life?” and “Do you now smoke cigarettes?” Drinking status was grouped as non-drinkers, moderate drinkers (0–15 g/day for women, 0–30 g/day for men), and heavy drinkers (≥ 15 g/day for women, ≥ 30 g/day for men). Participants were considered hypertensive if they reported a hypertension diagnosis, were taking antihypertensive medications, or had an average blood pressure ≥ 140/90 mmHg. Participants were defined as diabetic if they reported a diabetes diagnosis, were receiving oral hypoglycemic agents or insulin, had fasting glucose ≥ 7.0 mmol/L, or HbA1c ≥ 6.5%. CVD was identified based on a self-reported history of stroke, myocardial infarction, angina pectoris, coronary heart disease, or congestive heart failure.

### Statistical analysis

Baseline characteristics were presented as weighted means with standard errors and as sample sizes with weighted percentages for continuous and categorical variables, respectively. Group differences were analyzed with t-tests, chi-square tests, or Mann-Whitney U tests. Mediation analysis was performed to evaluate the mediating role of depression on the association of hearing loss with all-cause and cardiovascular mortality. Given the well-established dose–response relationships between hearing loss, depression, and mortality [25–28], we used the PTA as a continuous measure of hearing loss and the PHQ-9 score as a continuous indicator of depressive symptoms in the mediation analysis. Hearing loss was assessed across multiple categories, including speech-frequency hearing loss (SFHL), low-frequency hearing loss (LFHL), high-frequency hearing loss (HFHL), and hearing loss across multiple frequencies (0.5, 1, 2, 3, 4, 6, and 8 kHz). Following established mediation analysis principles [29, 30], three conditions were required: (1) PTA must be significantly associated with all-cause and cardiovascular mortality, and this relationship must be attenuated after including depression in the model, evaluated using weighted multivariate Cox proportional hazards regression models; (2) PTA must be significantly associated with depression, assessed via weighted multivariate linear regression models; and (3) depression must be significantly related to all-cause and cardiovascular mortality, also evaluated using weighted multivariate Cox regression models. If these conditions were met, mediation analyses were conducted to evaluate the mediating effect of depression on the association of hearing loss with all-cause and cardiovascular mortality. The significance and size of the mediation effect were tested using the Sobel test, with covariate adjustment to minimize potential confounding [29, 30]. All analyses accounted for the complex, multistage probability sampling design of NHANES, including sample weights, clustering, and stratification, as recommended by the NHANES analytic guidelines. For participants with hearing assessment data, we applied the 2-year MEC sample weights for each included cycle and adjusted them appropriately for pooled multi-cycle analysis. We performed multivariate imputation using chained equations (MICE) to address missing covariate data (Supplementary Table 1), generating 100 completed datasets via the R package mice. To combine results across imputations while accounting for the complex survey design, we applied Rubin’s rules [31, 32], which allowed for averaging point estimates and incorporating both within- and between-imputation variances. Additionally, as a sensitivity analysis, we repeated the mediation analysis using complete cases without imputation to evaluate the robustness of our findings. Statistical analyses were conducted using R software (version 4.4.1), with statistical significance defined as a two-sided P-value less than 0.05.

## Result

### Baseline characteristics

Table 1 presents the baseline characteristics of the study cohort, which included 9,827 participants, among whom 1,144 died from all causes. The participants had a mean age of 49.32 ± 0.42 years, with 4,967 (48.98%) males. The median follow-up duration was 70 months. Participants with elevated all-cause mortality risk were generally older, non-Hispanic White, former smokers, and moderate alcohol consumers, as well as having a history of hypertension, diabetes, and CVD. Additionally, comparable results were observed in the multiply imputed dataset, as shown in Supplementary Table 2, which presents the weighted baseline characteristics of participants after multiple imputation. Moreover, individuals who experienced all-cause mortality exhibited a significantly higher proportion of PHQ-9 scores exceeding 10 points, indicating a greater prevalence of depression compared to survivors. Additionally, a significant positive association was observed between SF-PTA, LF-PTA, HF-PTA, and all-cause mortality, highlighting the potential impact of hearing loss on mortality outcomes.

**Table 1:** Weighted characteristics of included participants, NHANES 2005 to 2018

Characteristics	Total	Non-all-cause mortality	All-cause mortality	*P* value
Total	*n* = 9827	*n* = 8683	*n* = 1144	
Age, year, Mean (SE)	49.32 (0.42)	47.37 (0.39)	71.64 (0.61)	< 0.001
Sex, n (%)				0.067
Male	4967 (48.98)	4303 (48.73)	664 (51.87)	
Female	4860 (51.01)	4380 (51.27)	480 (48.13)	
Race, n (%)				< 0.001
Mexican American	1292 (7.75)	1200 (8.08)	92 (3.95)	
Other Hispanic	972 (5.62)	932 (5.96)	40 (1.68)	
Non-Hispanic White	4049 (68.50)	3276 (67.28)	773 (82.48)	
Non-Hispanic Black	2215 (10.75)	2014 (10.87)	201 (9.48)	
Other Race	1299 (7.38)	1261 (7.81)	38 (2.41)	
BMI, kg/m^2^, Mean (SE)	29.19 (0.15)	29.22 (0.16)	28.80 (0.26)	0.135
Smoking status				< 0.001
None	5445 (55.14)	4992 (56.41)	453 (40.69)	
Former	2554 (26.38)	2049 (24.95)	505 (42.73)	
Current	1821 (18.48)	1636 (18.64)	185 (16.58)	
Drinking status				< 0.001
Non-drinker	1439 (12.70)	1263 (12.08)	176 (21.72)	
Moderate drinker	3353 (43.87)	2960 (43.16)	393 (54.23)	
Heavy drinker	3133 (43.43)	2977 (44.76)	156 (24.05)	
Hypertension, n (%)				< 0.001
No	5368 (60.98)	5045 (63.72)	323 (29.70)	
Yes	4350 (39.02)	3541 (36.28)	809 (70.30)	
Diabetes, n (%)				< 0.001
No	7806 (84.39)	7068 (85.91)	738 (66.97)	
Yes	2021 (15.61)	1615 (14.09)	406 (33.03)	
CVD, n (%)				< 0.001
No	8609 (90.40)	7871 (92.52)	738 (66.17)	
Yes	1218 (9.60)	812 (7.48)	406 (33.83)	
PHQ-9, points, n (%)	2.97 (0.08)	2.94 (0.08)	3.38 (0.23)	< 0.05
SF-PTA, dB, Mean (SE)	14.07 (0.34)	12.62 (0.30)	30.60 (0.68)	< 0.001
LF-PTA, dB, Mean (SE)	11.39 (0.30)	10.19 (0.27)	24.98 (0.66)	< 0.001
HF-PTA, dB, Mean (SE)	24.38 (0.51)	22.08 (0.46)	50.85 (1.03)	< 0.001

Values were presented as the weighted mean (standard errors) or sample numbers (weighted percentages). The differences between groups were assessed using the t-tests, chi-square tests, or Mann-Whitney U tests (P-value <0.05 indicates statistical significance)

*BMI* Body mass index, *CVD* Cardiovascular disease, *HF-PTA* High-frequency pure-tone average, *LF-PTA* Low-frequency pure-tone average, *NHANES* National Health and Nutrition Examination Survey, *PHQ-9* 9-item Patient Health Questionnaire, *SF-PTA* Speech-frequency pure-tone average

### Relationship between hearing loss and mortality

Table 2 presents the association of hearing loss with all-cause and cardiovascular mortality. After adjusting for confounding factors (Model 2), hearing loss showed a significant association with elevated all-cause and cardiovascular mortality. The hazard ratios (HRs) for all-cause mortality per 10 dB increase in SF-PTA, LF-PTA, and HF-PTA were 1.12 (95% CI: 1.07–1.17), 1.10 (95% CI: 1.05–1.16), and 1.10 (95% CI: 1.06–1.15), respectively. Similarly, the corresponding HRs for cardiovascular mortality were 1.14 (95% CI: 1.05–1.23), 1.13 (95% CI: 1.03–1.23), and 1.09 (95% CI: 1.03–1.16), respectively. These findings demonstrate a robust positive association of hearing loss with all-cause and cardiovascular mortality. Upon further adjustment for depression, an attenuation in HRs was observed, suggesting a potential mediating effect of depression on the association of hearing loss with all-cause and cardiovascular mortality. Specifically, the HRs for all-cause mortality associated with per 10 dB increase in SF-PTA, LF-PTA, and HF-PTA decreased to 1.11 (95% CI: 1.06–1.16), 1.09 (95% CI: 1.04–1.15), and 1.10 (95% CI: 1.05–1.14), respectively. Similarly, the adjusted HRs for cardiovascular mortality were 1.12 (95% CI: 1.04–1.21), 1.11 (95% CI: 1.02–1.22), and 1.08 (95% CI: 1.02–1.14), respectively. These results reinforce the hypothesis that depression may act as a significant mediator in the relationship between hearing loss and mortality. Consistent with these findings, frequency-specific analyses (Supplementary Table 3) further confirmed the significant positive correlation between hearing loss and mortality. Notably, a reduction in HR values was observed across all frequency categories after adjusting for depression, underscoring the potential mediating effect of depression.

**Table 2:** Association of hearing loss with all-cause and cardiovascular mortality

	Crude model		Model 1		Model 2		Model 3	
	HR (95%CI)	*P* value	HR (95%CI)	*P* value	HR (95%CI)	*P* value	HR (95%CI)	*P* value
All-cause mortality								
SF-PTA*	1.54 (1.48–1.61)	<0.001	1.13 (1.08–1.19)	<0.001	1.12 (1.07–1.17)	<0.001	1.11 (1.06–1.16)	<0.001
LF-PTA*	1.52 (1.47–1.57)	<0.001	1.12 (1.07–1.17)	<0.001	1.10 (1.05–1.16)	<0.001	1.09 (1.04–1.15)	<0.001
HF-PTA*	1.45 (1.42–1.49)	<0.001	1.11 (1.07–1.16)	<0.001	1.10 (1.06–1.15)	<0.001	1.10 (1.05–1.14)	<0.001
Cardiovascular mortality								
SF-PTA*	1.63 (1.53–1.73)	<0.001	1.16 (1.07–1.25)	<0.001	1.14 (1.05–1.23)	0.001	1.12 (1.04–1.21)	0.003
LF-PTA*	1.61 (1.52–1.70)	<0.001	1.15 (1.05–1.24)	0.002	1.13 (1.03–1.23)	0.008	1.11 (1.02–1.22)	<0.05
HF-PTA*	1.53 (1.48–1.58)	<0.001	1.10 (1.03–1.17)	0.005	1.09 (1.03–1.16)	0.004	1.08 (1.02–1.14)	0.006

Model 1: adjusted for age, sex, and race

Model 2: adjusted for model 1 + BMI, smoking status, drinking status, hypertension, diabetes, and CVD

Model 3: adjusted for model 2 + depression

*BMI* Body mass index, *CVD* Cardiovascular disease, *CI* Confidence interval, *HR* Hazard ratio, *HF-PTA* High-frequency pure-tone average, *LF-PTA* Low-frequency pure-tone average, *SF-PTA* Speech-frequency pure-tone average

*per 10 dB PTA increase

### Relationship between hearing loss and depression

The relationship between hearing loss and depression are presented in Table 3. In the cohort analyzing all-cause mortality, hearing loss was significantly and positively associated with depression. After adjusting for potential confounding factors (Model 2), the β values for per 10 dB increase in SF-PTA, LF-PTA, and HF-PTA were 0.25 (95% CI: 0.14–0.36), 0.22 (95% CI: 0.10–0.33), and 0.18 (95% CI: 0.10–0.25), respectively. Similarly, in the cohort analyzing cardiovascular mortality, a significant positive connection linking hearing loss to depression was observed. The β values for per 10 dB increase in SF-PTA, LF-PTA, and HF-PTA were 0.28 (95% CI: 0.15–0.40), 0.26 (95% CI: 0.13–0.38), and 0.18 (95% CI: 0.10–0.26), respectively. Furthermore, frequency-specific analyses (Supplementary Table 4) confirmed this trend, reinforcing the consistency of the association between hearing loss and depression across different hearing frequency ranges.

**Table 3:** Association between hearing loss and depression

	Crude model		Model 1		Model 2	
	β (95%CI)	*P* value	β (95%CI)	*P* value	β (95%CI)	*P* value
All-cause mortality cohort						
SF-PTA*	0.11 (0.03–0.20)	0.009	0.37 (0.25–0.49)	< 0.001	0.25 (0.14–0.36)	< 0.001
LF-PTA*	0.15 (0.05–0.25)	0.002	0.33 (0.20–0.46)	< 0.001	0.22 (0.10–0.33)	< 0.001
HF-PTA*	0.03 (0.00–0.05.00.05)	< 0.05	0.25 (0.17–0.34)	< 0.001	0.18 (0.10–0.25)	< 0.001
Cardiovascular mortality cohort						
SF-PTA*	0.15 (0.05–0.24)	0.003	0.41 (0.27–0.54)	< 0.001	0.28 (0.15–0.40)	< 0.001
LF-PTA*	0.20 (0.09–0.31)	< 0.001	0.38 (0.24–0.52)	< 0.001	0.26 (0.13–0.38)	< 0.001
HF-PTA*	0.04 (0.01–0.07)	< 0.05	0.26 (0.17–0.35)	< 0.001	0.18 (0.10–0.26)	< 0.001

Model 1: adjusted for age, sex, and race

Model 2: adjusted for model 1 + BMI, smoking status, drinking status, hypertension, diabetes, and CVD

*β* Regression coefficient, *BMI* Body mass index, *CVD* Cardiovascular disease, *CI* Confidence interval, *HF-PTA* High-frequency pure-tone average, *LF-PTA* Low-frequency pure-tone average, *SF-PTA* Speech-frequency pure-tone average

*per 10 dB PTA increase

### Relationship between depression and mortality

Table 4 displays the association between depression and all-cause as well as cardiovascular mortality. After adjusting for confounding factors (Model 2), depression showed a significant correlation with higher mortality risk. The HRs of depression for all-cause and cardiovascular mortality were 1.05 (95% CI: 1.03–1.07) and 1.06 (95% CI: 1.04–1.08), respectively. Further adjustments for SF-PTA (Model 3), LF-PTA (Model 4), and HF-PTA (Model 5) resulted in HRs for all-cause mortality of 1.05 (95% CI: 1.03–1.06), 1.05 (95% CI: 1.03–1.06), and 1.05 (95% CI: 1.03–1.07), respectively. Similarly, the HRs for cardiovascular mortality were 1.06 (95% CI: 1.04–1.07), 1.06 (95% CI: 1.04–1.07), and 1.06 (95% CI: 1.04–1.09), all of which remained statistically significant. Moreover, associations between depression and all-cause as well as cardiovascular mortality persisted when adjusting for hearing thresholds at frequencies from 500 Hz to 8000 Hz (Supplementary Table 5). These findings indicate that depression acts as an independent risk factor for all-cause as well as cardiovascular mortality, further underscoring the critical impact of mental health on overall survival outcomes.

**Table 4:** Association of depression with all-cause and cardiovascular mortality

	HR (95%CI)	*P* value
All-cause mortality		
Crude model	1.04 (1.02–1.06)	<0.001
Model 1	1.07 (1.05–1.08)	<0.001
Model 2	1.05 (1.03–1.07)	<0.001
Model 3	1.05 (1.03–1.06)	<0.001
Model 4	1.05 (1.03–1.06)	<0.001
Model 5	1.05 (1.03–1.07)	<0.001
Cardiovascular mortality		
Crude model	1.05 (1.02–1.08)	<0.001
Model 1	1.07 (1.05–1.09)	<0.001
Model 2	1.06 (1.04–1.08)	<0.001
Model 3	1.06 (1.04–1.07)	<0.001
Model 4	1.06 (1.04–1.07)	<0.001
Model 5	1.06 (1.04–1.09)	<0.001

Model 1: adjusted for age, sex, and race

Model 2: adjusted for model 1 + BMI, smoking status, drinking status, hypertension, diabetes, and CVD

Model 3: adjusted for model 2 + SF-PTA

Model 4: adjusted for model 2 + LF-PTA

*BMI* Body mass index, *CVD* Cardiovascular disease, *CI* Confidence interval, *HR* Hazard ratio, *HF-PTA* High-frequency pure-tone average, *LF-PTA* Low-frequency pure-tone average, *SF-PTA* Speech-frequency pure-tone average

### Mediation analysis

**Fig. 2:**
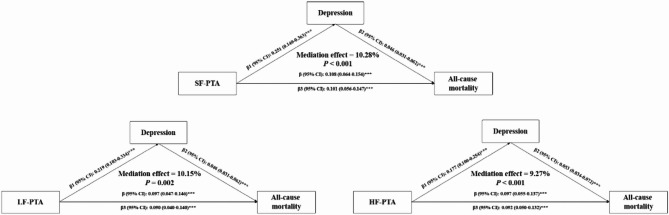
Mediation models illustrating the role of depression in the association of hearing loss with all-cause mortality. Adjusted for age, sex, race, BMI, smoking status, drinking status, hypertension, diabetes, and CVD.*:*P*<0.05; **: *P*<0.01; ***: *P*<0.001. Indirect effect: β1*β2; Direct effect: β3; Total effect: β. Abbreviations: β, regression coefficient; BMI, body mass index; CVD, cardiovascular disease; CI, confidence interval; HF-PTA, high-frequency pure-tone average; LF-PTA, low-frequency pure-tone average; SF-PTA, speech-frequency pure-tone average

The mediating role of depression on the association of hearing loss with all-cause mortality is shown in Fig. 2. In the connection between hearing loss and all-cause mortality, depression exhibited significant mediation effects across the SF-PTA, LF-PTA, and HF-PTA groups, with mediation proportions of 10.28%, 10.15%, and 9.27%, respectively.

**Fig. 3:**
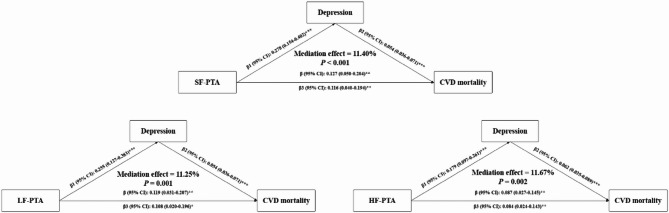
Mediation models illustrating the role of depression in the association of hearing loss with cardiovascular mortality. Adjusted for age, sex, race, BMI, smoking status, drinking status, hypertension, diabetes, and CVD.*:*P*<0.05; **: *P*<0.01; ***: *P*<0.001. Indirect effect: β1*β2; Direct effect: β3; Total effect: β. Abbreviations: β, regression coefficient; BMI, body mass index; CVD, cardiovascular disease; CI, confidence interval; HF-PTA, high-frequency pure-tone average; LF-PTA, low-frequency pure-tone average; SF-PTA, speech-frequency pure-tone average

The mediating role of depression on the association of hearing loss with cardiovascular mortality is shown in Fig. 3. Similarly, in the connection between hearing loss and cardiovascular mortality, depression also demonstrated significant mediation effects, accounting for 11.40%, 11.25%, and 11.67% of the relationship in the SF-PTA, LF-PTA, and HF-PTA groups, respectively.

Additionally, in the complete case sensitivity analysis, depression continued to show a significant mediating effect on the associations between hearing loss and both all-cause and cardiovascular mortality. The estimated proportions mediated were comparable to, or slightly higher than, those derived from the imputed datasets (Supplementary Table 6). Furthermore, significant and stable mediation effects of depression were observed on the association of hearing loss at various frequencies with all-cause and cardiovascular mortality, as detailed in Supplementary Table 7. For all-cause mortality, the mediation effect of depression across hearing frequencies from 500 Hz to 8000 Hz ranged from 7.13% to 11.19%, while for cardiovascular mortality, it ranged from 7.49% to 16.38%. These mediation effects remained within a stable range and were all statistically significant. These results strongly support the mediating role of depression in the effect of hearing loss on mortality risk.

Discussion.

This study demonstrates the significant mediating effect of depression on the association of hearing loss with all-cause and cardiovascular mortality. Specifically, depression mediated the associations of SFHL, LFHL, and HFHL with all-cause mortality by 10.28%, 10.15%, and 9.27%, respectively. Similarly, for cardiovascular mortality, depression accounted for 11.40%, 11.25%, and 11.67% of the associations in the SFHL, LFHL, and HFHL groups, respectively. In addition to these frequency-specific categories, consistent and significant mediation effects were also observed across individual hearing frequencies ranging from 0.5 to 8 kHz, further reinforcing the robustness of the findings. These findings underscore the profound influence of mental health on all-cause and cardiovascular mortality outcomes in individuals with hearing loss, emphasizing the critical need for integrating depression detection and interventions into hearing loss management strategies to mitigate mortality risk.

Depression remains a global mental health challenge and is the leading cause of disability related to mental health conditions [33]. It is strongly associated with suicide and premature death due to other diseases [34], and its link to all-cause and cardiovascular mortality has been well established [14, 15, 35]. Given its high prevalence and frequent recurrence, depression imposes a substantial economic burden globally [33, 36]. As understanding of depression deepens, its risk factors have been increasingly identified, with hearing loss emerging as a critical contributor [6, 7, 37]. In this study, we reaffirm the robust connection between hearing loss and depression, consistent with findings from prior research. Several studies utilizing the NHANES database have similarly demonstrated a significant association between hearing loss and self-reported depression [8, 38]. Additionally, UK Biobank studies have linked hearing loss to elevated risks of both depression and anxiety [39]. Research from Chinese cohorts also supports this association, revealing significant connection of self-reported hearing loss with depressive symptoms [40, 41]. Furthermore, a nationwide Korean cohort study confirmed a bidirectional association connecting hearing loss and depression [42]. These findings collectively demonstrate the consistent positive relationship between hearing loss and depression across diverse populations and cultural contexts.

The pathways through which hearing loss leads to depression remain incompletely understood. Psychosocial, functional, and neurobiological mechanisms are likely involved [7]. Within the World Health Organization’s International Classification of Functioning, Disability and Health framework, impairments in auditory function can lead to activity limitations in communication and participation restrictions in social roles, which together reduce health-related quality of life. These restrictions create conditions in which depressive symptoms can emerge and persist [43]. Communication barriers in daily settings increase listening effort and cognitive load, leading to persistent fatigue and diminished psychosocial well-being, both of which are proximal determinants of poorer quality of life and depressive symptoms [44]. Limitations in work and community roles add to this burden; workers with hearing loss show a greater need for recovery after work and higher job stress, classic participation restrictions that undermine well-being and can precipitate or aggravate depressive affect [45]. A substantial body of evidence also links hearing loss with social isolation and loneliness, key psychosocial stressors strongly related to depression in later life [46]. Hearing loss poses a substantial risk of depressive symptoms in older adults, particularly among those facing socioeconomic disadvantage [47]. In addition, hearing loss is associated with decrements in physical function. Systematic reviews and cohort studies report slower gait speed, poorer balance, and higher fall risk, which further restrict daily activities and independence and reinforce a cycle of quality-of-life decline and emotional distress [48–50]. Meanwhile, hearing loss-induced reductions in central auditory input can trigger compensatory neural changes [51], disrupted auditory-limbic connectivity [52], and frontal atrophy [53], ultimately impairing emotional regulation and increasing the risk of depression [54].

Beyond the positive correlation of hearing loss with depression, this study also reinforces the established link between hearing loss and increased all-cause and cardiovascular mortality. In a nationally representative American cohort, moderate and severe hearing loss was related to a 54% higher mortality risk after adjusting for age, with a clear dose-response relationship observed [10]. Research analyzing data from the National Health Interview Survey found that hearing loss is a significant independent predictor of lifespan, independent of other well-determined determinants of mortality [55]. A systematic review of 26 observational studies involving over 1.2 million participants further confirmed that every 30-dB increase in hearing thresholds doubled the relative risk of mortality [9]. Conversely, adults with hearing loss who regularly used hearing aids had a reduced mortality risk compared to non-users [11]. Several mechanisms could underlie the link between hearing loss and mortality, including shared comorbidities, socioeconomic status, marital status, frailty, mental health disorders, and safety hazards [9]. Among these, mental health disorders, particularly depression, remain underexplored in terms of their contribution to mortality risk.

With population aging worldwide, the prevalence of hearing loss, particularly age-related hearing loss (ARHL), is increasing rapidly [1]. Both hearing impairment and its associated mortality have become pressing public health challenges [2, 3]. Understanding the mechanisms linking hearing loss to all-cause and cardiovascular mortality is essential for developing effective, targeted interventions aimed at reducing these risks. Our study contributes novel evidence that depression significantly mediates the connection between hearing loss and mortality outcomes, including both all-cause and cardiovascular mortality, across speech-frequency, low-frequency, high-frequency, and multi-frequency hearing loss categories. These findings highlight the critical need to integrate mental health assessments and interventions into routine hearing care practices. Unfortunately, despite the well-documented connection between hearing loss and depression, mental health assessments are often overlooked in audiological care [56]. At the same time, the quality-of-life impact of hearing-related disability is a principal and modifiable pathway to depression in people with hearing loss, yet it remains under-recognized and seldom addressed in routine audiology services [47, 57]. This implementation gap sustains depressive burden and may contribute to downstream excess mortality. Integrating standardized quality-of-life assessments with validated depression screeners such as the Patient Health Questionnaire-9 and the Geriatric Depression Scale is therefore both urgent and necessary in hearing clinics [24, 58]. A quality-of-life-oriented care pathway that combines routine depression screening and timely mental health care with comprehensive communication-strategy training and programs that preserve functional capacity and social participation can alleviate activity limitations, sustain role functioning, and mitigate depressive symptoms. Embedding these assessments within hearing evaluations enables clinicians to deliver integrated, patient-centered care that addresses social participation and mental health needs. Such integration is expected to improve quality of life, alleviate depressive symptoms, and may reduce mortality among people with hearing loss, with meaningful clinical and public-health implications.

Based on a large-scale prospective cohort study, our findings provide robust evidence supporting the hypothesis that depression significantly mediates the association of hearing loss with all-cause and cardiovascular mortality. Notably, this is the first study to systematically demonstrate the mediating effects of depression across diverse hearing loss categories, including SFHL, LFHL, HFHL, and hearing loss spanning multiple frequencies. This comprehensive analysis offers valuable insights into the pivotal role of depression as a mediator on the association of hearing loss with mortality outcomes. These results highlight the importance of routine assessment of quality of life and implementation of psychological health screening in individuals with hearing loss to facilitate early detection and timely intervention for depression. Such integrated care approaches may contribute to reducing the risks of both all-cause and cardiovascular mortality in this vulnerable population. However, several limitations warrant consideration. First, while depression accounts for a significant portion of the link between hearing loss and mortality, the potential roles of other mediators remain unexplored and merit further investigation to fully elucidate the underlying mechanisms. Second, using self-reported questionnaires to evaluate depression may lead to recall bias, potentially compromising data accuracy. Third, this study did not differentiate hearing loss by etiology, limiting the ability to assess whether depression’s mediating role varies across different causes of hearing loss. Fourth, because hearing-assistive devices can improve communication, enhance quality of life, and reduce depressive symptoms, the absence of reliable data on device use and adherence may have introduced residual confounding. Information on hearing-aid or cochlear-implant use was missing or inconsistently collected across the included NHANES cycles; therefore, we could not adjust for device use. Future cohorts should incorporate standardized measures of device uptake and adherence to evaluate their influence on these pathways. Finally, as a multifactorial condition, hearing loss is influenced by genetic predisposition and environmental exposures. Although we adjusted for numerous confounders, residual confounding, such as genetic predisposition, may still influence the findings.

Conclusion.

This large-scale prospective cohort study provides robust evidence that depression significantly mediates the association of hearing loss with all-cause and cardiovascular mortality. The mediating role of depression is significant and stable across various hearing loss categories, including speech-frequency, low-frequency, high-frequency, and multi-frequency impairments. These results emphasize the profound impact of depression on adverse health outcomes in individuals with hearing loss, underscoring the critical need to incorporate routine depression screening and interventions into standard audiological care. Early detection and targeted interventions for depression may offer an effective strategy to reduce the elevated risks of all-cause and cardiovascular mortality associated with hearing impairment, ultimately improving patient outcomes.

Supplementary Information.

## Supplementary Information

Supplementary Material 1: Supplementary Table 1. Variables subjected to multiple imputation and corresponding missing data proportions. Supplementary Material 2: Supplementary Table 2. Weighted characteristics of participants based on multiply imputed data. Supplementary Material 3: Supplementary Table 3. Association of hearing loss at various frequencies with all-cause and cardiovascular mortality. Supplementary Material 4: Supplementary Table 4. Association between hearing loss at various frequencies and depression. Supplementary Material 5: Supplementary Table 5. Association of depression with all-cause and cardiovascular mortality. Supplementary Material 6: Supplementary Table 6. Mediation analysis of depression in the association of hearing loss with all-cause and cardiovascular mortality in complete cases. Supplementary Material 7: Supplementary Table 7. Mediation analysis of depression in the association of hearing loss at various frequencies with all-cause and cardiovascular mortality.

## Data Availability

The datasets generated and/or analyzed during the current study are available in the [National Health and Nutrition Examination Survey], [https://www.cdc.gov/nchs/nhanes/].

## Abbreviations

ARHL: Age-related hearing loss
β: Regression coefficient
BMI: Body mass index
CI: Confidence interval
CVD: Cardiovascular disease
dB: Decibel
GDS: Geriatric Depression Scale
HFHL: High-frequency hearing loss
HF-PTA: High-frequency pure-tone average
HR: Hazard ratio
ICD-10: International Classification of Diseases, 10th Revision
LFHL: Low-frequency hearing loss
LF-PTA: Low-frequency pure-tone average
NCHS: National Center for Health Statistics
NHANES: National Health and Nutrition Examination Survey
PHQ-9: 9-item Patient Health Questionnaire
SFHL: Speech-frequency hearing loss
SF-PTA: Speech-frequency pure-tone average
