# Supplementary material for: The mediating effect of depression on the association of hearing loss with all-cause and cardiovascular mortality: a prospective cohort study
Source: BMC Public Health. 2025 Nov 25;25:4133. doi: 10.1186/s12889-025-25489-9 (PMC12649039; doi:10.1186/s12889-025-25489-9)
Supplement: Supplementary file 1 — Supplementary Material 1: Supplementary Table 1. Variables subjected to multiple imputation and corresponding missing data proportions. Supplementary Material 2: Supplementary Table 2. Weighted characteristics of participants based on multiply imputed data. Supplementary Material 3: Supplementary Table 3. Association of hearing loss at various frequencies with all-cause and cardiovascular mortality. Supplementary Material 4: Supplementary Table 4. Association between hearing loss at various frequencies and depression. Supplementary Material 5: Supplementary Table 5. Association of depression with all-cause and cardiovascular mortality. Supplementary Material 6: Supplementary Table 6. Mediation analysis of depression in the association of hearing loss with all-cause and cardiovascular mortality in complete cases. Supplementary Material 7: Supplementary Table 7. Mediation analysis of depression in the association of hearing loss at various frequencies with all-cause and cardiovascular mortality. [file 12889_2025_25489_MOESM1_ESM.docx]

# The mediating effect of depression on the association of hearing loss with all-cause and cardiovascular mortality: A prospective cohort study

**Shaoyan Zhang^1†^, Haohong Lai ^2†^, Chunling Liu^1*^**

^1^Department of Otorhinolaryngology Head and Neck Surgery, the People's Hospital of Baoan Shenzhen, China.

^2^Department of Otolaryngology, Sun Yat-sen Memorial Hospital, Sun Yat-sen University, 107th Yanjiangxi Road, Guangzhou 510120, Guangdong, China.

^†^Equal contribution and first authorship

*** Correspondence：**

Chunling Liu

Email: **ChunlingLiuSZ@163.com**

**Supplementary Table 1** Variables subjected to multiple imputation and corresponding missing data proportions

| **Covariate** | **Number of missing values** | **Percentage missing (%)** |
| --- | --- | --- |
| BMI | 78 | 0.79 |
| Smoking status | 7 | 0.07 |
| Drinking status | 1902 | 19.35 |
| Hypertension | 109 | 1.11 |

**Supplementary Table 2** Weighted characteristics of participants based on multiply imputed data

| Characteristics | Total | Non-all-cause mortality | All-cause mortality | *P* value |
| --- | --- | --- | --- | --- |
| Total | n=9827 | n=8683 | n=1144 |  |
| Age, year, Mean (SE) | 49.32 (0.42) | 47.37 (0.39) | 71.64 (0.61) | <0.001 |
| Sex, n (%) |  |  |  | 0.067 |
| Male | 4967 (48.98) | 4303 (48.73) | 664 (51.87) |  |
| Female | 4860 (51.01) | 4380 (51.27) | 480 (48.13) |  |
| Race, n (%) |  |  |  | <0.001 |
| Mexican American | 1292 (7.75) | 1200 (8.08) | 92 (3.95) |  |
| Other Hispanic | 972 (5.62) | 932 (5.96) | 40 (1.68) |  |
| Non-Hispanic White | 4049 (68.50) | 3276 (67.28) | 773 (82.48) |  |
| Non-Hispanic Black | 2215 (10.75) | 2014 (10.87) | 201 (9.48) |  |
| Other Race | 1299 (7.38) | 1261 (7.81) | 38 (2.41) |  |
| BMI, kg/m^2^, Mean (SE) | 29.13 (0.15) | 29.18 (0.16) | 28.63 (0.26) | 0.056 |
| Smoking status |  |  |  | <0.001 |
| None | 5452 (55.16) | 4998 (56.42) | 454 (40.71) |  |
| Former | 2554 (26.37) | 2049 (24.94) | 505 (42.71) |  |
| Current | 1821 (18.47) | 1636 (18.64) | 185 (16.58) |  |
| Drinking status |  |  |  | <0.001 |
| Non-drinker | 1470 (10.88) | 1286 (10.50) | 184 (15.24) |  |
| Moderate drinker | 3733 (40.05) | 3256 (39.81) | 477 (42.67) |  |
| Heavy drinker | 4624 (49.07) | 4141 (49.69) | 483 (42.09) |  |
| Hypertension, n (%) |  |  |  | <0.001 |
| No | 5477 (61.33) | 5142 (64.05) | 335 (30.20) |  |
| Yes | 4350 (38.67) | 3541 (35.95) | 809 (69.80) |  |
| Diabetes, n (%) |  |  |  | <0.001 |
| No | 7806 (84.39) | 7068 (85.91) | 738 (66.97) |  |
| Yes | 2021 (15.61) | 1615 (14.09) | 406 (33.03) |  |
| CVD, n (%) |  |  |  | <0.001 |
| No | 8609 (90.40) | 7871 (92.52) | 738 (66.17) |  |
| Yes | 1218 (9.60) | 812 (7.48) | 406 (33.83) |  |
| PHQ-9, points, n (%) | 2.97 (0.08) | 2.94 (0.08) | 3.38 (0.23) | <0.05 |
| SF-PTA, dB, Mean (SE) | 14.07 (0.34) | 12.62 (0.30) | 30.60 (0.68) | <0.001 |
| LF-PTA, dB, Mean (SE) | 11.39 (0.30) | 10.19 (0.27) | 24.98 (0.66) | <0.001 |
| HF-PTA, dB, Mean (SE) | 24.38 (0.51) | 22.08 (0.46) | 50.85 (1.03) | <0.001 |

Values were presented as the weighted mean (standard errors) or sample numbers (weighted percentages). The differences between groups were assessed using the t-tests, chi-square tests, or Mann-Whitney U tests (P-value <0.05 indicates statistical significance).

Abbreviations: BMI, body mass index; CVD, cardiovascular disease; HF-PTA, high-frequency pure-tone average; LF-PTA, low-frequency pure-tone average; NHANES, National Health and Nutrition Examination Survey; PHQ-9, 9-item Patient Health Questionnaire; SF-PTA, speech-frequency pure-tone average.

**Supplementary Table 3** Association of hearing loss at various frequencies with all-cause and cardiovascular mortality

|  | **Crude model** | |  | **Model 1** | |  | **Model 2** | |  | **Model 3** | |
| --- | --- | --- | --- | --- | --- | --- | --- | --- | --- | --- | --- |
|  | **HR (95%CI)** | ***P* value** |  | **HR (95%CI)** | ***P* value** |  | **HR (95%CI)** | ***P* value** |  | **HR (95%CI)** | ***P* value** |
| **All-cause mortality** | | | | | | | | | | | |
| 500Hz* | 1.52 (1.46-1.59) | ＜0.001 |  | 1.15 (1.10-1.21) | ＜0.001 |  | 1.13 (1.07-1.19) | ＜0.001 |  | 1.12 (1.06-1.18) | ＜0.001 |
| 1000Hz* | 1.44 (1.38-1.50) | ＜0.001 |  | 1.09 (1.05-1.14) | ＜0.001 |  | 1.08 (1.04-1.13) | ＜0.001 |  | 1.08 (1.03-1.12) | 0.001 |
| 2000Hz* | 1.41 (1.35-1.46) | ＜0.001 |  | 1.06 (1.02-1.11) | 0.005 |  | 1.06 (1.02-1.10) | 0.004 |  | 1.05 (1.01-1.10) | 0.009 |
| 3000Hz* | 1.41 (1.36-1.45) | ＜0.001 |  | 1.09 (1.05-1.13) | ＜0.001 |  | 1.08 (1.04-1.11) | ＜0.001 |  | 1.07 (1.03-1.11) | ＜0.001 |
| 4000Hz* | 1.40 (1.36-1.44) | ＜0.001 |  | 1.10 (1.07-1.14) | ＜0.001 |  | 1.09 (1.05-1.13) | ＜0.001 |  | 1.08 (1.04-1.12) | ＜0.001 |
| 6000Hz* | 1.39 (1.35-1.44) | ＜0.001 |  | 1.09 (1.04-1.14) | ＜0.001 |  | 1.08 (1.04-1.13) | ＜0.001 |  | 1.07 (1.03-1.12) | ＜0.001 |
| 8000Hz* | 1.39 (1.36-1.43) | ＜0.001 |  | 1.09 (1.05-1.13) | ＜0.001 |  | 1.08 (1.04-1.12) | ＜0.001 |  | 1.07 (1.03-1.12) | ＜0.001 |
| **Cardiovascular mortality** | | | | | | | | | | | |
| 500Hz* | 1.64 (1.53-1.76) | ＜0.001 |  | 1.20 (1.11-1.30) | ＜0.001 |  | 1.17 (1.07-1.28) | ＜0.001 |  | 1.16 (1.06-1.26) | 0.001 |
| 1000Hz* | 1.51 (1.42-1.61) | ＜0.001 |  | 1.12 (1.04-1.21) | 0.003 |  | 1.11 (1.03-1.20) | 0.009 |  | 1.10 (1.02-1.19) | ＜0.05 |
| 2000Hz* | 1.47 (1.40-1.56) | ＜0.001 |  | 1.07 (1.00-1.14) | ＜0.05 |  | 1.07 (1.00-1.14) | ＜0.05 |  | 1.06 (0.99-1.13) | 0.095 |
| 3000Hz* | 1.48 (1.42-1.55) | ＜0.001 |  | 1.09 (1.03-1.16) | 0.002 |  | 1.08 (1.03-1.14) | 0.004 |  | 1.08 (1.02-1.13) | 0.007 |
| 4000Hz* | 1.47 (1.42-1.53) | ＜0.001 |  | 1.10 (1.05-1.16) | ＜0.001 |  | 1.09 (1.04-1.14) | ＜0.001 |  | 1.08 (1.03-1.14) | 0.001 |
| 6000Hz* | 1.46 (1.40-1.52) | ＜0.001 |  | 1.07 (1.00-1.14) | ＜0.05 |  | 1.07 (1.00-1.13) | ＜0.05 |  | 1.06 (0.99-1.13) | 0.082 |
| 8000Hz* | 1.46 (1.41-1.52) | ＜0.001 |  | 1.06 (1.10-1.12) | ＜0.05 |  | 1.06 (1.01-1.12) | ＜0.05 |  | 1.06 (1.00-1.12) | ＜0.05 |

* per 10 dB hearing threshold increase

Model 1: adjusted for age, sex, and race;

Model 2: adjusted for model 1 + BMI, smoking status, drinking status, hypertension, diabetes, and CVD;

Model 3: adjusted for model 2 + depression.

Abbreviations: BMI, body mass index; CVD, cardiovascular disease; CI, confidence interval; HR, hazard ratio.

**Supplementary Table 4** Association between hearing loss at various frequencies and depression

|  | **β (95%CI)** | ***P* value** |
| --- | --- | --- |
| **All-cause mortality** **cohort** | | |
| 500Hz | 0.19 (0.08-0.30) | <0.001 |
| 1000Hz | 0.19 (0.08-0.30) | <0.001 |
| 2000Hz | 0.15 (0.07-0.24) | <0.001 |
| 3000Hz | 0.13 (0.06-0.21) | <0.001 |
| 4000Hz | 0.18 (0.11-0.24) | <0.001 |
| 6000Hz | 0.17 (0.10-0.23) | <0.001 |
| 8000Hz | 0.11 (0.05-0.18) | <0.001 |
| **Cardiovascular mortality** **cohort** | | |
| 500Hz | 0.22 (0.09-0.36) | <0.001 |
| 1000Hz | 0.22 (0.10-0.34) | <0.001 |
| 2000Hz | 0.17 (0.08-0.27) | <0.001 |
| 3000Hz | 0.14 (0.06-0.22) | <0.001 |
| 4000Hz | 0.18 (0.11-0.24) | <0.001 |
| 6000Hz | 0.17 (0.10-0.24) | <0.001 |
| 8000Hz | 0.11 (0.05-0.18) | <0.001 |

Adjusted for age, sex, race, BMI, smoking status, drinking status, hypertension, diabetes, and CVD.

Abbreviations: β, regression coefficient; BMI, body mass index; CVD, cardiovascular disease; CI, confidence interval.

**Supplementary Table 5** Association of depression with all-cause and cardiovascular mortality

|  | **HR (95%CI)** | ***P* value** |
| --- | --- | --- |
| **All-cause mortality** | | |
| 500Hz | 1.05 (1.03-1.06) | ＜0.001 |
| 1000Hz | 1.05 (1.03-1.06) | ＜0.001 |
| 2000Hz | 1.05 (1.03-1.06) | ＜0.001 |
| 3000Hz | 1.05 (1.03-1.06) | ＜0.001 |
| 4000Hz | 1.05 (1.03-1.06) | ＜0.001 |
| 6000Hz | 1.05 (1.04-1.07) | ＜0.001 |
| 8000Hz | 1.05 (1.03-1.08) | ＜0.001 |
| **Cardiovascular mortality** | | |
| 500Hz | 1.05 (1.04-1.07) | ＜0.001 |
| 1000Hz | 1.06 (1.04-1.07) | ＜0.001 |
| 2000Hz | 1.06 (1.04-1.08) | ＜0.001 |
| 3000Hz | 1.06 (1.04-1.08) | ＜0.001 |
| 4000Hz | 1.06 (1.04-1.07) | ＜0.001 |
| 6000Hz | 1.07 (1.04-1.09) | ＜0.001 |
| 8000Hz | 1.06 (1.04-1.09) | ＜0.001 |

Adjusted for age, sex, race, BMI, smoking status, drinking status, hypertension, diabetes, CVD, and hearing thresholds at various frequencies.

Abbreviations: BMI, body mass index; CVD, cardiovascular disease; CI, confidence interval; HR, hazard ratio.

**Supplementary Table 6** Mediation analysis of depression in the association of hearing loss with all-cause and cardiovascular mortality in complete cases

|  | β1 (95%CI) | β2 (95%CI) | β3 (95%CI) | β (95%CI) | Proportion | *P* value |
| --- | --- | --- | --- | --- | --- | --- |
| **All-cause mortality cohort** | | | | | | |
| SF-PTA | 0.235 (0.115-0.355) *** | 0.055 (0.035-0.075)*** | 0.101 (0.056-0.145)*** | 0.104 (0.058-0.150)*** | 12.09% | 0.002 |
| LF-PTA | 0.200 (0.076-0.323)** | 0.055 (0.035-0.076)*** | 0.088 (0.039-0.138)*** | 0.091 (0.040-0.141)*** | 11.81% | 0.005 |
| HF-PTA | 0.175 (0.090-0.260)*** | 0.054 (0.034-0.074)*** | 0.087 (0.047-0.128)*** | 0.093 (0.052-0.134)*** | 9.97% | 0.001 |
| **Cardiovascular mortality cohort** | | | | | | |
| SF-PTA | 0.256 (0.120-0.391)*** | 0.068 (0.039-0.097)*** | 0.107 (0.030-0.185)** | 0.110 (0.032-0.188)** | 15.36% | 0.003 |
| LF-PTA | 0.230 (0.089-0.370)** | 0.068 (0.039-0.097)*** | 0.097 (0.008-0.186)* | 0.100 (0.010-0.189)* | 15.33% | 0.007 |
| HF-PTA | 0.176 (0.085-0.267)*** | 0.068 (0.040-0.096)*** | 0.074 (0.015-0.133)* | 0.077 (0.018-0.136)** | 15.18% | 0.003 |

Adjusted for age, sex, race, BMI, smoking status, drinking status, hypertension, diabetes, and CVD.

*: *P* <0.05; **: *P* <0.01; ***: *P* <0.001.

Indirect effect: β1*β2; Direct effect: β3; Total effect: β.

Abbreviations: β, regression coefficient; BMI, body mass index; CVD, cardiovascular disease; CI, confidence interval. HF-PTA, high-frequency pure-tone average; LF-PTA, low-frequency pure-tone average; SF-PTA, speech-frequency pure-tone average.

**Supplementary Table 7** Mediation analysis of depression in the association of hearing loss at various frequencies with all-cause and cardiovascular mortality

|  | β1 (95%CI) | β2 (95%CI) | β3 (95%CI) | β (95%CI) | Proportion | *P* value |
| --- | --- | --- | --- | --- | --- | --- |
| **All-cause mortality** | | | | | | |
| 500Hz | 0.189 (0.075-0.303) *** | 0.046 (0.031-0.061)*** | 0.113 (0.062-0.166)*** | 0.122 (0.070-0.173)*** | 7.13% | 0.005 |
| 1000Hz | 0.191 (0.081-0.300) *** | 0.047 (0.031-0.062)*** | 0.073 (0.029-0.117)** | 0.079 (0.036-0.122)*** | 10.87% | 0.003 |
| 2000Hz | 0.152 (0.065-0.240) *** | 0.048 (0.032-0.063)*** | 0.053 (0.013-0.093)** | 0.058 (0.018-0.097)** | 11.99% | 0.003 |
| 3000Hz | 0.134 (0.061-0.207) *** | 0.047 (0.032-0.062)*** | 0.068 (0.031-0.103)*** | 0.073 (0.037-0.107)*** | 8.49% | 0.002 |
| 4000Hz | 0.176 (0.112-0.239) *** | 0.046 (0.032-0.061)*** | 0.079 (0.044-0.113)*** | 0.086 (0.051-0.120)*** | 9.37% | <0.001 |
| 6000Hz | 0.168 (0.103-0.233) *** | 0.053 (0.035-0.071)*** | 0.071 (0.030-0.112)*** | 0.077 (0.036-0.118)*** | 11.14% | <0.001 |
| 8000Hz | 0.113 (0.048-0.177) *** | 0.053 (0.034-0.072)*** | 0.073 (0.035-0.111)*** | 0.078 (0.039-0.116)*** | 7.62% | 0.004 |
| **Cardiovascular mortality** | | | | | | |
| 500Hz | 0.224 (0.092-0.355) *** | 0.053 (0.036-0.069)*** | 0.146 (0.057-0.233)** | 0.159 (0.071-0.247)*** | 7.49% | 0.003 |
| 1000Hz | 0.223 (0.103-0.343) *** | 0.054 (0.036-0.071)*** | 0.094 (0.015-0.171)* | 0.103 (0.025-0.181)** | 11.28% | 0.002 |
| 2000Hz | 0.173 (0.078-0.268) *** | 0.056 (0.039-0.073)*** | 0.057 (-0.010-0.122) | 0.065 (0.000-0.129)* | 14.44% | 0.002 |
| 3000Hz | 0.135 (0.056-0.215) *** | 0.056 (0.039-0.072)*** | 0.073 (0.019-0.125)** | 0.078 (0.026-0.131)** | 9.32% | 0.003 |
| 4000Hz | 0.177 (0.111-0.243) *** | 0.055 (0.038-0.072)*** | 0.079 (0.031-0.127)** | 0.086 (0.038-0.134)*** | 11.01% | <0.001 |
| 6000Hz | 0.171 (0.103-0.240) *** | 0.065 (0.042-0.089)*** | 0.057 (-0.007-0.122) | 0.064 (0.001-0.126)* | 16.38% | <0.001 |
| 8000Hz | 0.113 (0.045-0.181) *** | 0.062 (0.036-0.089)*** | 0.058 (0.004-0.112)* | 0.060 (0.005-0.114)* | 10.82% | 0.008 |

Adjusted for age, sex, race, BMI, smoking status, drinking status, hypertension, diabetes, and CVD.

*: *P* <0.05; **: *P* <0.01; ***: *P* <0.001.

Indirect effect: β1*β2; Direct effect: β3; Total effect: β.

Abbreviations: β, regression coefficient; BMI, body mass index; CVD, cardiovascular disease; CI, confidence interval.
